# Effects of GRP78 on Endoplasmic Reticulum Stress and Inflammatory Response in Macrophages of Large Yellow Croaker (*Larimichthys crocea*)

**DOI:** 10.3390/ijms24065855

**Published:** 2023-03-20

**Authors:** Jie Sun, Kangsen Mai, Qinghui Ai

**Affiliations:** 1 Key Laboratory of Aquaculture Nutrition and Feed (Ministry of Agriculture and Rural Affairs), Key Laboratory of Mariculture (Ministry of Education), Ocean University of China, 5 Yushan Road, Qingdao 266003, China; 2 Laboratory for Marine Fisheries Science and Food Production Processes, Qingdao National Laboratory for Marine Science and Technology, 1 Wenhai Road, Qingdao 266237, China

**Keywords:** ER stress, inflammation, GRP78, palmitic acid, large yellow croaker

## Abstract

Endoplasmic reticulum (ER) homeostasis plays a vital role in cell physiological functions. Various factors can destroy the homeostasis of the ER and cause ER stress. Moreover, ER stress is often related to inflammation. Glucose-regulated protein 78 (GRP78) is an ER chaperone, which plays a vital role in maintaining cellular homeostasis. Nevertheless, the potential effects of GRP78 on ER stress and inflammation is still not fully elucidated in fish. In the present study, ER stress and inflammation was induced by tunicamycin (TM) or palmitic acid (PA) in the macrophages of large yellow croakers. GRP78 was treated with an agonist/inhibitor before or after the TM/PA treatment. The results showed that the TM/PA treatment could significantly induce ER stress and an inflammatory response in the macrophages of large yellow croakers whereas the incubation of the GRP78 agonist could reduce TM/PA-induced ER stress and an inflammatory response. Moreover, the incubation of the GRP78 inhibitor could further induce TM/PA-induced ER stress and an inflammatory response. These results provide an innovative idea to explain the relationship between GRP78 and TM/PA-induced ER stress or inflammation in large yellow croakers.

## 1. Introduction

The endoplasmic reticulum (ER) is essential for regulating calcium storage, protein folding, and the biosynthesis of lipids, steroids, and carbohydrates [1,2]. Cellular stress conditions such as exposure to free radicals, a glucose deficiency, and the accumulation of misfolded or unfolded proteins can cause ER stress and activate an unfolded protein response (UPR) [3,4,5,6]. One of the most essential protective mechanisms induced by the UPR is the upregulation of the expression of glucose-regulated protein 78 (GRP78).

GRP78 is also commonly referred to as BiP, the immunoglobulin heavy chain binding protein, which belongs to the heat shock protein 70 (HSP70) family. In non-stressed cells, GRP78 is bound to all three UPR transmembrane stress sensors, which are maintained in an inactive state, including double-stranded RNA-dependent protein kinase-like ER kinase (PERK), activating transcription factor 6 (ATF6), and inositol-requiring protein 1α (IRE1α). Upon ER stress, all three sensors become active after being released from GRP78. The UPR active form then downregulates the translation of protein and increases the correct folding [7].

All three UPR sensors have been found to regulate the inflammatory response [8,9,10,11]; for example, to activate TNFα and NF-κB. GRP78 also has been found to regulate inflammation. GRP78 is known as part of the resolution-associated molecular patterns (RAMPs) family that can be anti-inflammatory and promote the resolution of inflammation [12,13,14]. GRP78 regulates FAT10 expression by modulating the NF-κB pathway; to be more specific, the activation of the NF-κB pathway can increase the expression of FAT10, which is a gene counteracting the tumor suppressor p53 [15]. An antibody directly against the GRP78 carboxyl terminal domain can inhibit the pro-proliferative NF-κB signaling cascade in cancer cells [16].

Tunicamycin (TM) is a common ER stress inducer. In previous studies, the results showed that TM incubation could significantly induce ER stress-related genes [17,18,19,20]. Meanwhile, a TM treatment has often induced the expression of proinflammatory genes [19,21,22,23]. Palmitic acid (PA) is known as one of the most common saturated fatty acids, which has also been demonstrated to induce the inflammation of macrophages in mammals [24]. Palm oil (PO) is enriched with PA and is widely used as a substitute for fish oil in aquaculture [25]. Previous studies have shown that the overuse of PO often induces inflammation [26,27]; meanwhile, PA has been revealed to induce inflammation and ER stress [28,29,30]. However, the relationship between GRP78 and TM/PA-induced inflammation or ER stress is still not completely understood.

Although fish are less evolved than mammals, their immune response system and nutrient-sensing are evolutionarily conservative [31]. The large yellow croaker (*Larimichthys crocea*) is a vital fish-farming species in China. In recent years, PO has been widely used in large yellow croaker feed as a promising alternative to fish oil. The large yellow croaker can be used as a terrific experimental model animal. Hence, this study aimed to discover how GRP78 mediates ER stress and the inflammatory response in large yellow croakers. This study may develop an understanding of the molecular mechanism of ER stress and the inflammatory response mediated by GRP78.

## 2. Results

### 2.1. TM Treatment Induces ER Stress and an Inflammatory Response in the Macrophages of Large Yellow Croakers

**Figure 1:**
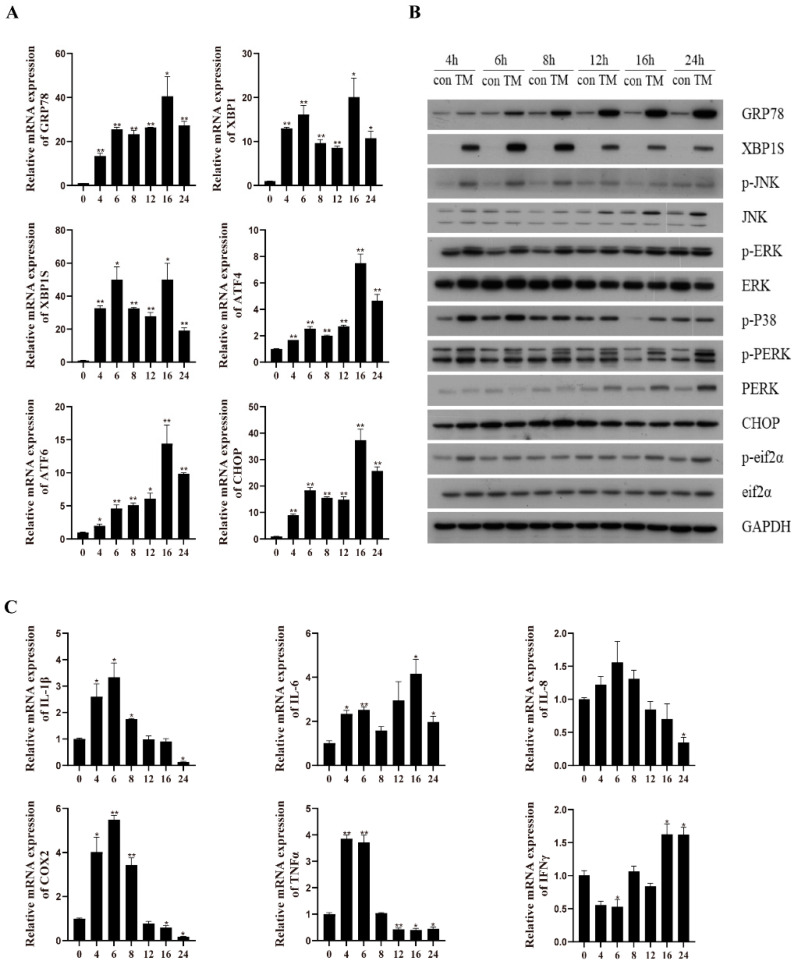
Effects of 1 μM TM treatment on ER stress and inflammation in macrophages of large yellow croakers. (**A**) Relative mRNA expression of ER stress-related genes after TM treatment for different time points. (**B**) Protein levels of ER stress-related proteins and inflammatory-related proteins after TM treatment for different time points. (**C**) Relative mRNA expression of inflammatory genes after TM treatment for different time points. Values are presented as mean ± SEM (*n* = 3). * Significant difference (*p* < 0.05); ** highly significant difference (*p* < 0.01).

The results indicated that TM incubation for different time points significantly upregulated the mRNA expression of *grp78*, *xbp1*, *xbp1s*, *chop*, *atf6*, and *atf4* compared with the control group (*p* < 0.05) (Figure 1A). Additionally, the protein levels of XBP1s and GRP78 as well as the phosphorylation levels of PERK and eif2α were significantly higher than those in the control group (Figure 1B) whereas the protein level of CHOP was not remarkably different. We then analyzed the effect of ER stress on inflammation in the macrophages. TM incubation significantly increased the mRNA levels of proinflammatory genes, including *il-8*, *il-6*, *il-1β*, *cox2*, *tnfα*, and *ifnγ* (*p* < 0.05) (Figure 1C). Moreover, TM incubation significantly upregulated the phosphorylation levels of JNK, ERK, and p38 (Figure 1B). These results demonstrated that the TM treatment could induce ER stress and an inflammatory response in the macrophages of large yellow croakers.

### 2.2. BiP Inducer X (GRP78 Agonist) Treatment Induces ER Stress and an Inflammatory Response in Macrophages

**Figure 2:**
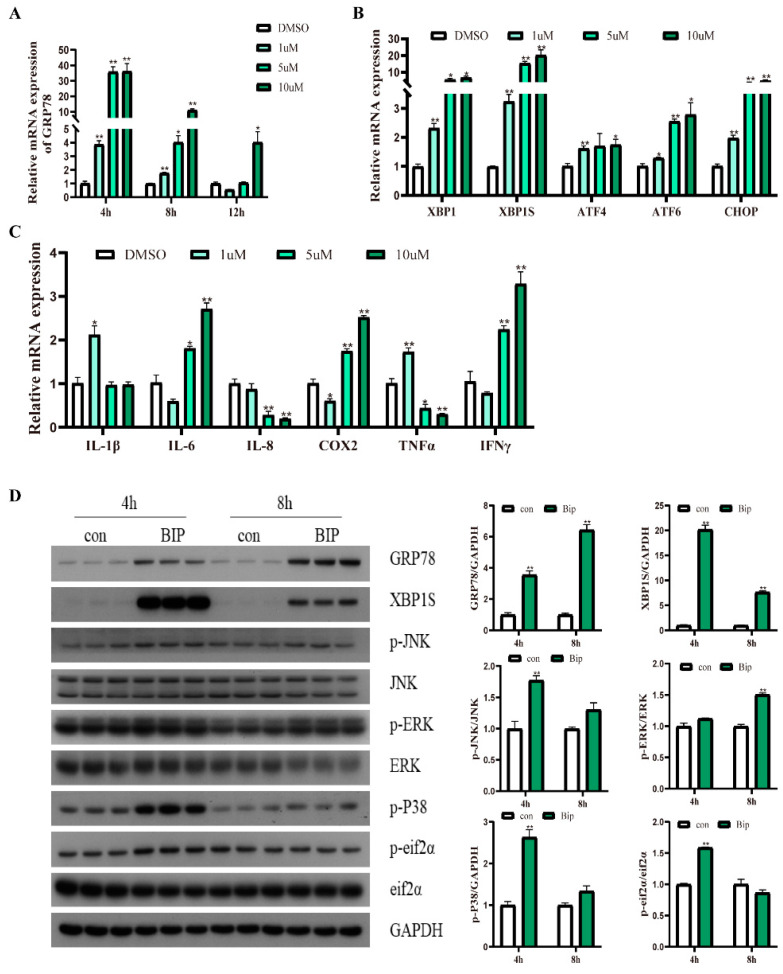
Effects of GRP78 agonist (BiP inducer X (BiP)) treatment on ER stress and inflammation in macrophages of large yellow croakers. (**A**) Relative mRNA expression of GRP78 after BiP treatment for different time points with different concentrations. (**B**) Relative mRNA expression of ER stress-related genes after BiP treatment for 4 h with different concentrations. (**C**) Relative mRNA expression of inflammatory genes after BiP treatment for 4 h with different concentrations. (**D**) Protein levels of ER stress-related proteins and inflammatory-related proteins after 10 μM BiP treatment for 4 h and 8 h. Values are presented as mean ± SEM (*n* = 3). * Significant difference (*p* < 0.05); ** highly significant difference (*p* < 0.01).

The results showed that BiP inducer X (BiP) treatments at different concentrations significantly upregulated the mRNA expression of *grp78* at 4 h and 8 h; however, at 12 h, only the 10 μM BiP incubation significantly upregulated the mRNA expression of *grp78* (*p* < 0.05) (Figure 2A). Moreover, the BiP treatments for different concentrations at 4 h significantly upregulated the mRNA expression of *xbp1*, *xbp1s*, *chop*, *atf6*, and *atf4* compared with the control group (*p* < 0.05) (Figure 2B). Additionally, the 10 μM BiP incubation for 4 h and 8 h significantly upregulated the protein levels of XBP1s and GRP78, and at 4 h significantly upregulated the phosphorylation levels of eif2α (*p* < 0.05) (Figure 2D). Different concentrations of the BiP treatment for 4 h significantly increased the mRNA levels of proinflammatory genes, including *il-6*, *il-1β*, *cox2*, *tnfα*, and *ifnγ* (*p* < 0.05) (Figure 2C). Furthermore, BiP incubation significantly upregulated the phosphorylation levels of JNK, ERK, and p38 (*p* < 0.05) (Figure 2D). These results indicated that the BiP treatment could induce ER stress and an inflammatory response in the macrophages of large yellow croakers.

### 2.3. HM03 (GRP78 Inhibitor) Treatment Induces ER Stress and an Inflammatory Response in Macrophages

**Figure 3:**
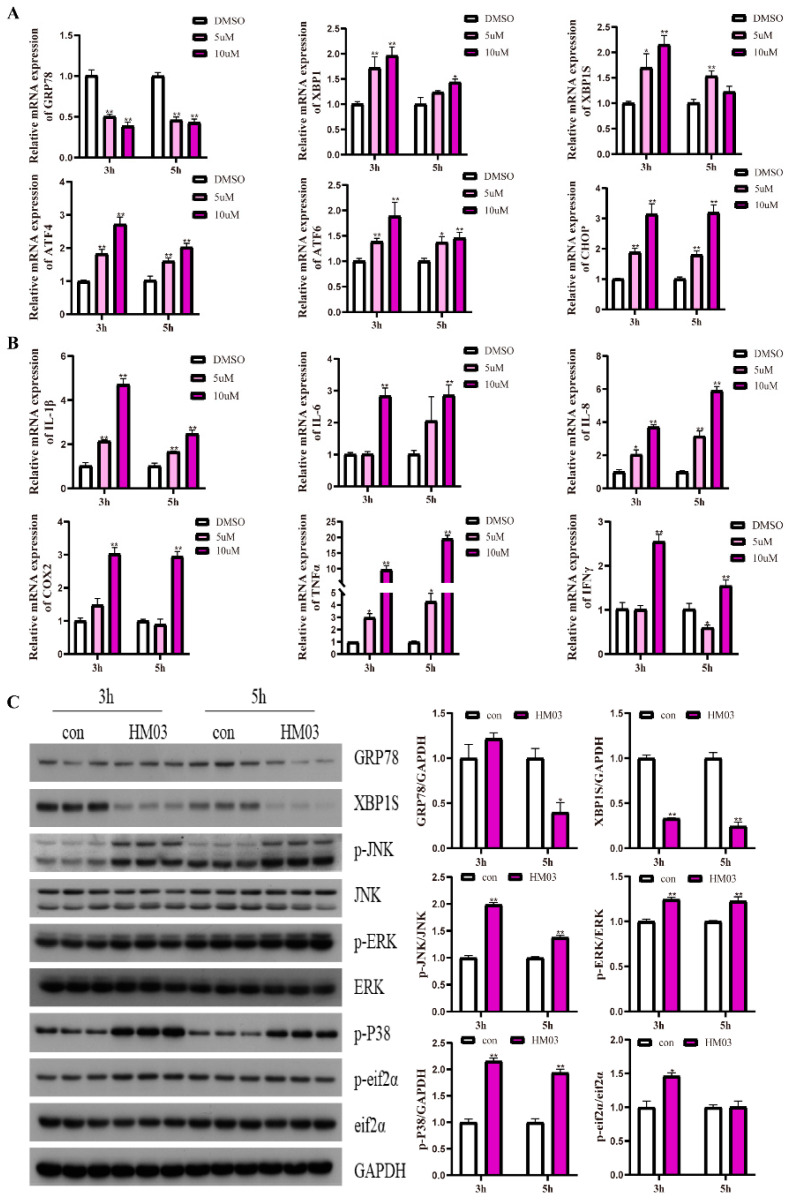
Effects of GRP78 inhibitor (HM03) treatment on ER stress and inflammation in macrophages of large yellow croakers. (**A**) Relative mRNA expression of ER stress-related genes after HM03 treatment for 3 h and 5 h with different concentrations. (**B**) Relative mRNA expression of inflammatory genes after HM03 treatment for 3 h and 5 h with different concentrations. (**C**) Protein levels of ER stress-related proteins and inflammatory-related proteins after 10 μM HM03 treatment for 3 h and 5 h. Values are presented as mean ± SEM (*n* = 3). * Significant difference (*p* < 0.05); ** highly significant difference (*p* < 0.01).

The results indicated that HM03 incubations for different concentrations significantly decreased the mRNA level of *grp78* at 3 h and 5 h (*p* < 0.05) (Figure 3A). HM03 incubation significantly upregulated the mRNA expression of *xbp1*, *xbp1s*, *chop*, *atf6*, and *atf4* compared with the control group (*p* < 0.05) (Figure 3A). Additionally, the 10 μM HM03 incubation for 5 h significantly decreased the protein level of GRP78 (*p* < 0.05) (Figure 3C). Moreover, HM03 incubation significantly decreased the protein level of XBP1s and upregulated the phosphorylation level of eif2α at 3 h (*p* < 0.05) (Figure 3C). The 10 μM HM03 incubation significantly increased the mRNA levels of proinflammatory genes, including *il-1β*, *il-6*, *il-8*, *cox2*, *tnfα*, and *ifnγ* (*p* < 0.05) (Figure 3B). Furthermore, HM03 incubation significantly upregulated the phosphorylation levels of JNK, ERK, and p38 (*p* < 0.05) (Figure 3C). These results demonstrated that the HM03 treatment could induce ER stress and an inflammatory response in the macrophages of large yellow croakers.

### 2.4. Effect of GRP78 on TM-Induced ER Stress and Inflammation in Macrophages

**Figure 4:**
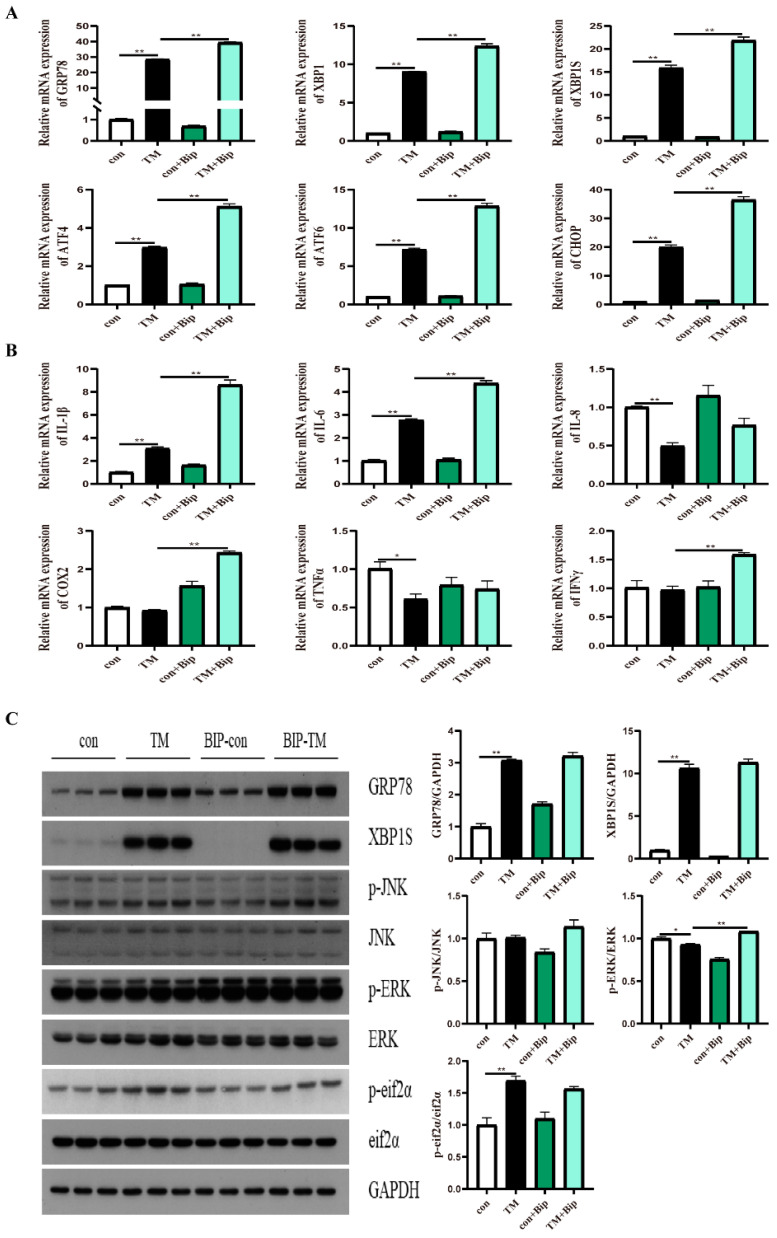
Effects of 10 μM BiP treatment for 4 h before 1 μM TM incubation for 16 h on ER stress and inflammation in macrophages of large yellow croakers. (**A**) Relative mRNA expression of ER stress-related genes. (**B**) Relative mRNA expression of inflammatory genes. (**C**) Protein levels of ER stress-related proteins and inflammatory-related proteins. Values are presented as mean ± SEM (*n* = 3). * Significant difference (*p* < 0.05); ** highly significant difference (*p* < 0.01).

**Figure 5:**
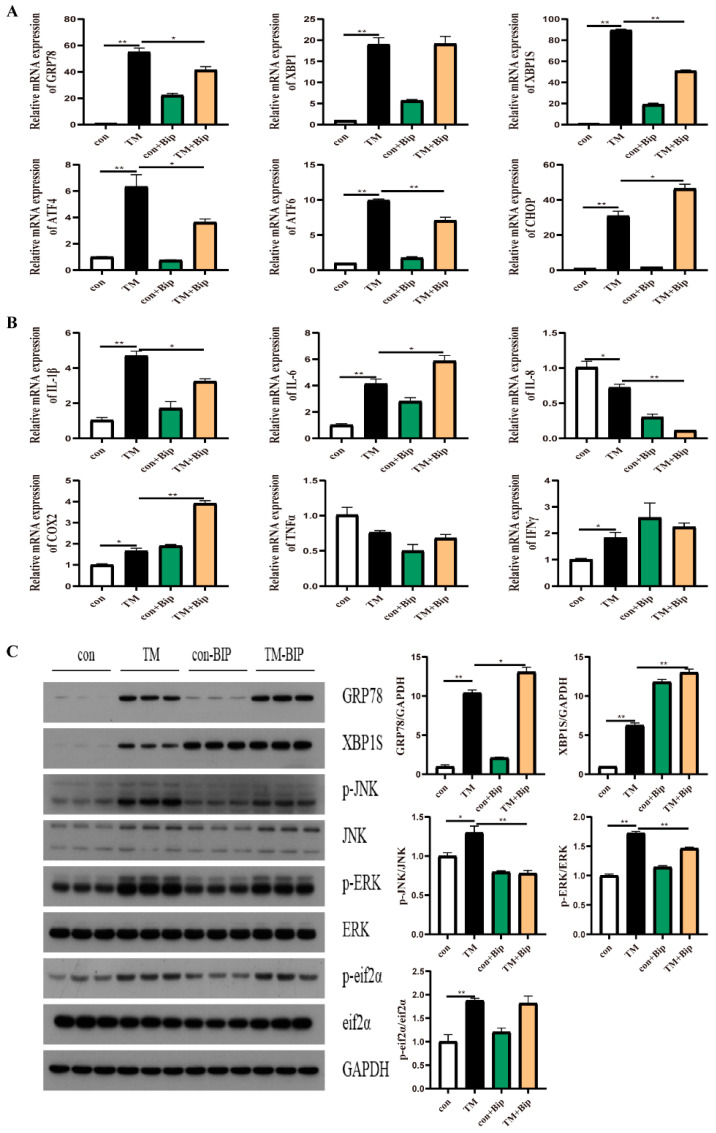
Effects of 10 μM BiP treatment for 4 h after 1 μM TM incubation for 16 h on ER stress and inflammation in macrophages of large yellow croakers. (**A**) Relative mRNA expression of ER stress-related genes. (**B**) Relative mRNA expression of inflammatory genes. (**C**) Protein levels of ER stress-related proteins and inflammatory-related proteins. Values are presented as mean ± SEM (*n* = 3). * Significant difference (*p* < 0.05); ** highly significant difference (*p* < 0.01).

**Figure 6:**
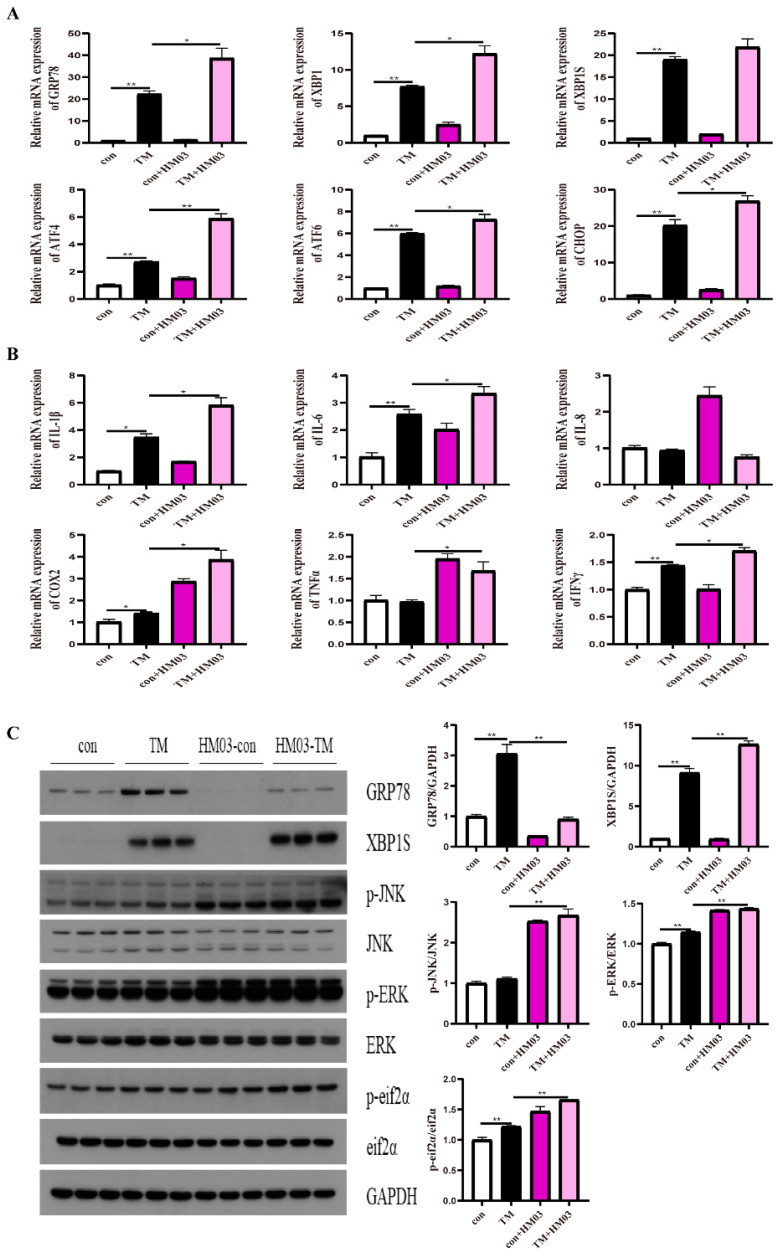
Effects of 10 μM HM03 treatment for 4 h before 1 μM TM incubation for 16 h on ER stress and inflammation in macrophages of large yellow croakers. (**A**) Relative mRNA expression of ER stress-related genes. (**B**) Relative mRNA expression of inflammatory genes. (**C**) Protein levels of ER stress-related proteins and inflammatory-related proteins. Values are presented as mean ± SEM (*n* = 3). * Significant difference (*p* < 0.05); ** highly significant difference (*p* < 0.01).

**Figure 7:**
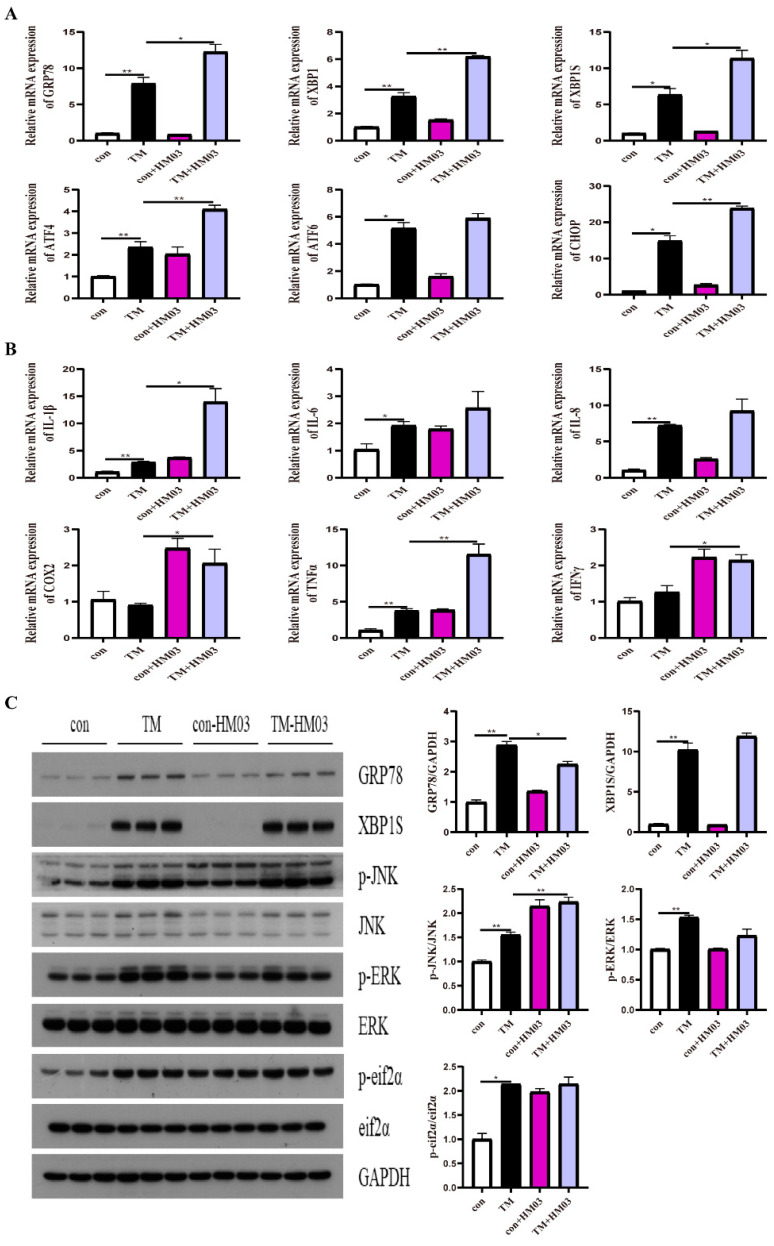
Effects of 10 μM HM03 treatment for 4 h after 1 μM TM incubation for 16 h on ER stress and inflammation in macrophages of large yellow croakers. (**A**) Relative mRNA expression of ER stress-related genes. (**B**) Relative mRNA expression of inflammatory genes. (**C**) Protein levels of ER stress-related proteins and inflammatory-related proteins. Values are presented as mean ± SEM (*n* = 3). * Significant difference (*p* < 0.05); ** highly significant difference (*p* < 0.01).

To confirm the effect of GRP78 on TM-induced ER stress and inflammation, 10 μM BiP and HM03 were incubated for 4 h before or after 1 μM TM incubation for 16 h. The results showed that the BiP treatment before TM incubation significantly upregulated the mRNA expression of *grp78*, *xbp1*, *xbp1s*, *chop*, *atf6*, and *atf4* compared with the TM treatment group (*p* < 0.05) (Figure 4A). Moreover, the proinflammatory genes, including *il-6*, *il-1β*, *cox2*, and *ifnγ*, were significantly increased (*p* < 0.05) (Figure 4B). BiP incubation significantly upregulated the phosphorylation level of ERK (*p* < 0.05) (Figure 4C). The BiP treatment after TM incubation significantly downregulated the mRNA expression of *grp78*, *xbp1s*, *atf4*, and *atf6* compared with the TM treatment group (*p* < 0.05) (Figure 5A). Additionally, the proinflammatory genes, including *il-8* and *il-1β*, were significantly downregulated (*p* < 0.05) (Figure 5B). Furthermore, the phosphorylation levels of JNK and ERK were significantly decreased (*p* < 0.05) (Figure 5C). The HM03 treatment before TM incubation significantly upregulated the mRNA expression of *grp78*, *xbp1*, *chop*, *atf6*, and *atf4* compared with the TM treatment group (*p* < 0.05) (Figure 6A). Moreover, the proinflammatory genes, including *il-6*, *il-1β*, *cox2*, *tnfα*, and *ifnγ,* were significantly increased (*p* < 0.05) (Figure 6B). Additionally, the protein level of XBP1s and the phosphorylation levels of JNK, ERK, and eif2α were significantly upregulated (*p* < 0.05) (Figure 6C). The HM03 treatment after TM incubation significantly upregulated the mRNA expression of *grp78*, *xbp1*, *xbp1s*, *chop*, and *atf4* compared with the TM treatment group (*p* < 0.05) (Figure 7A). Moreover, the proinflammatory genes, including *il-1β*, *cox2*, *ifnγ*, and *tnfα*, were significantly upregulated (*p* < 0.05) (Figure 7B). Furthermore, the phosphorylation level of JNK was significantly increased (*p* < 0.05) (Figure 7C). These results demonstrated that the BiP treatment after TM incubation could reduce the TM-induced ER stress and inflammatory response whereas the HM03 treatment could further induce the TM-induced ER stress and inflammatory response in the macrophages of large yellow croakers.

### 2.5. PA Treatment Induces ER Stress and an Inflammatory Response in Macrophages

**Figure 8:**
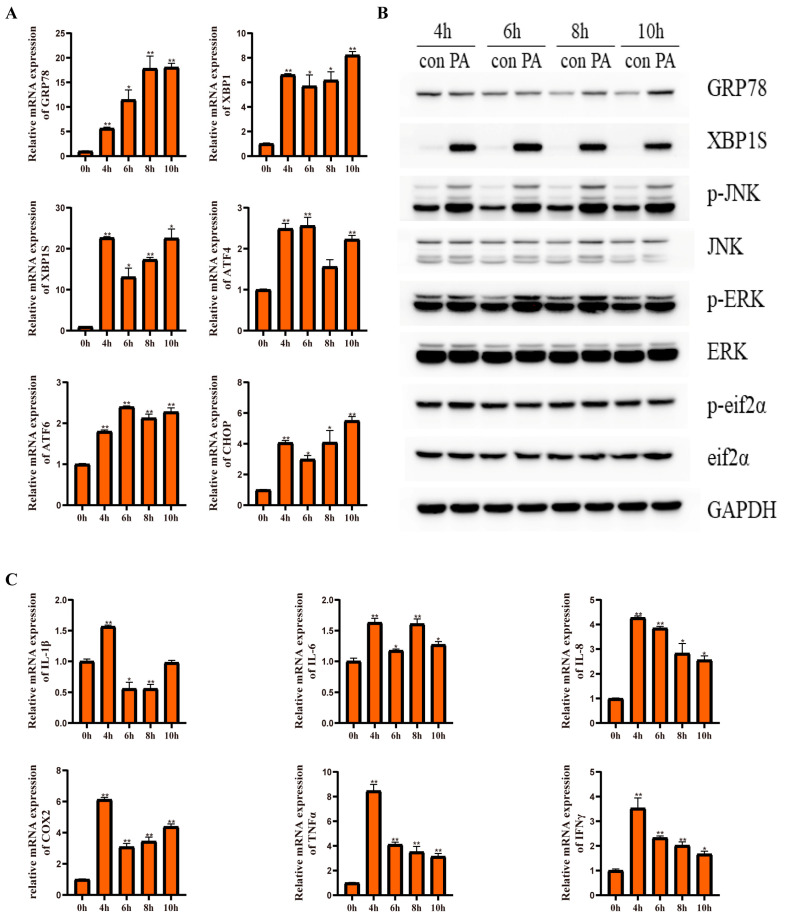
Effects of 500 μM PA treatment on ER stress and inflammation in macrophages of large yellow croakers. (**A**) Relative mRNA expression of ER stress-related genes after PA treatment for different time points. (**B**) Protein levels of ER stress-related proteins and inflammatory-related proteins after PA treatment for different time points. (**C**) Relative mRNA expression of inflammatory genes after PA treatment for different time points. Values are presented as mean ± SEM (*n* = 3). * Significant difference (*p* < 0.05); ** highly significant difference (*p* < 0.01).

The results indicated that PA incubation for different time points significantly upregulated the mRNA expression of *grp78*, *xbp1*, *xbp1s*, *chop*, *atf6*, and *atf4* compared with the control group (*p* < 0.05) (Figure 8A). Additionally, the protein levels of XBP1s and GRP78 were significantly upregulated compared with those in the control group (Figure 8B). Moreover, PA incubation significantly increased the mRNA levels of proinflammatory genes, including *il-1β*, *il-6*, *il-8*, *cox2*, *tnfα*, and *ifnγ* (*p* < 0.05) (Figure 8C). Furthermore, PA incubation significantly upregulated the phosphorylation levels of JNK and ERK (Figure 8B). These results indicated that the PA treatment could induce ER stress and an inflammatory response in the macrophages of large yellow croakers.

### 2.6. Effect of GRP78 on PA-Induced ER Stress and Inflammation in Macrophages

**Figure 9:**
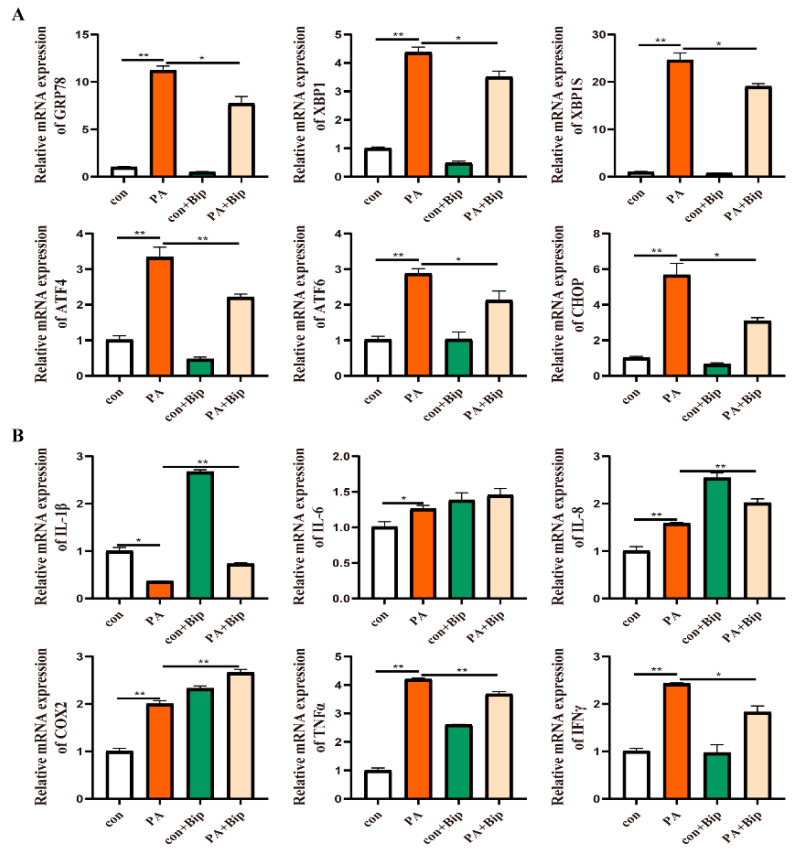
Effects of 10 μM BiP treatment for 4 h before 500 μM PA incubation for 6 h on ER stress and inflammation in macrophages of large yellow croakers. (**A**) Relative mRNA expression of ER stress-related genes. (**B**) Relative mRNA expression of inflammatory genes. Values are presented as mean ± SEM (*n* = 3). * Significant difference (*p* < 0.05); ** highly significant difference (*p* < 0.01).

**Figure 10:**
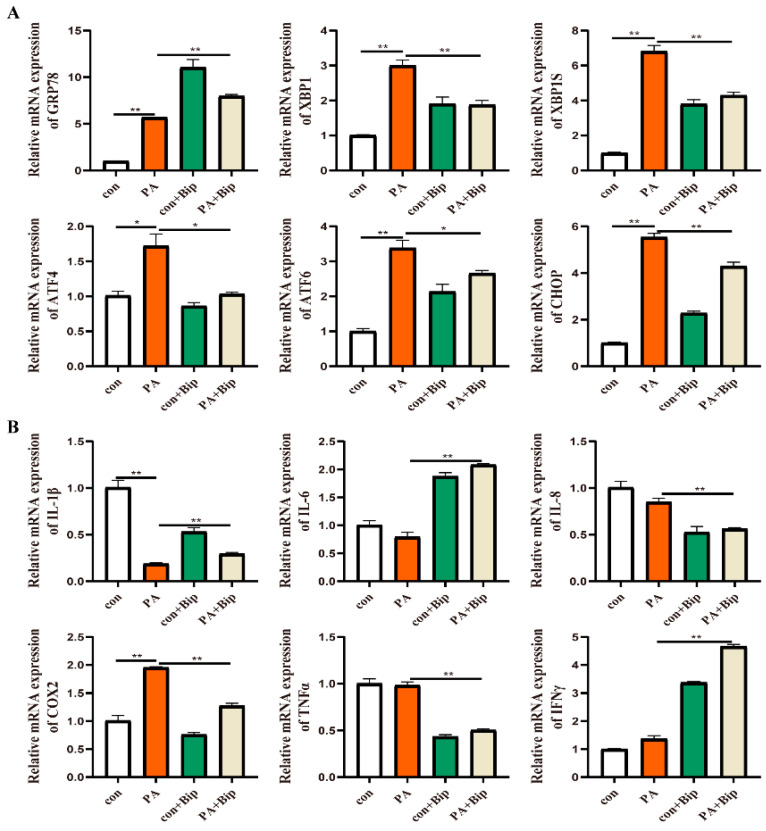
Effects of 10 μM BiP treatment for 4 h after 500 μM PA incubation for 6 h on ER stress and inflammation in macrophages of large yellow croakers. (**A**) Relative mRNA expression of ER stress-related genes. (**B**) Relative mRNA expression of inflammatory genes. Values are presented as mean ± SEM (*n* = 3). * Significant difference (*p* < 0.05); ** highly significant difference (*p* < 0.01).

**Figure 11:**
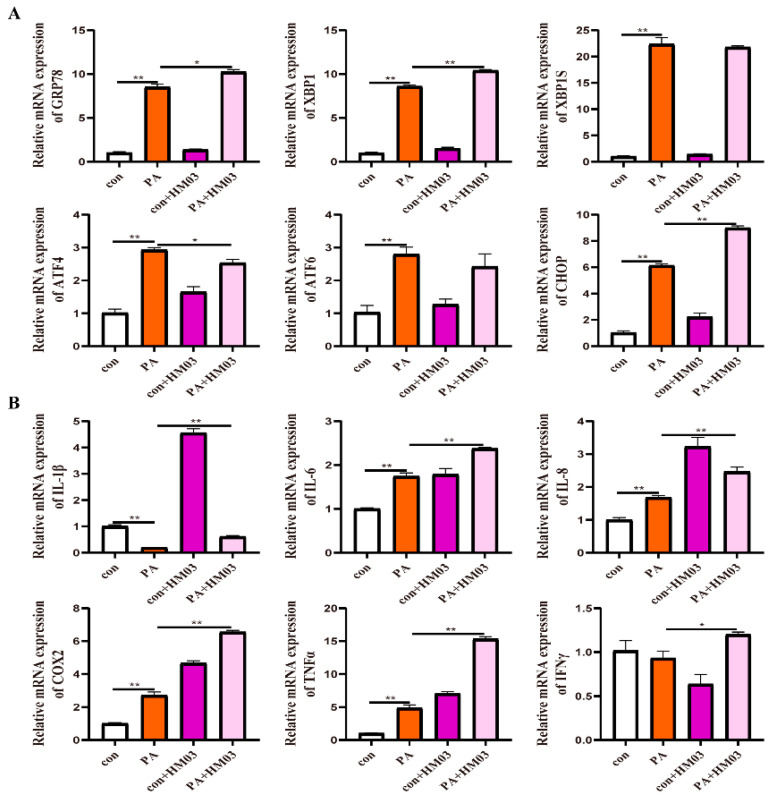
Effects of 10 μM HM03 treatment for 4 h before 500 μM PA incubation for 6 h on ER stress and inflammation in macrophages of large yellow croakers. (**A**) Relative mRNA expression of ER stress-related genes. (**B**) Relative mRNA expression of inflammatory genes. Values are presented as mean ± SEM (*n* = 3). * Significant difference (*p* < 0.05); ** highly significant difference (*p* < 0.01).

**Figure 12:**
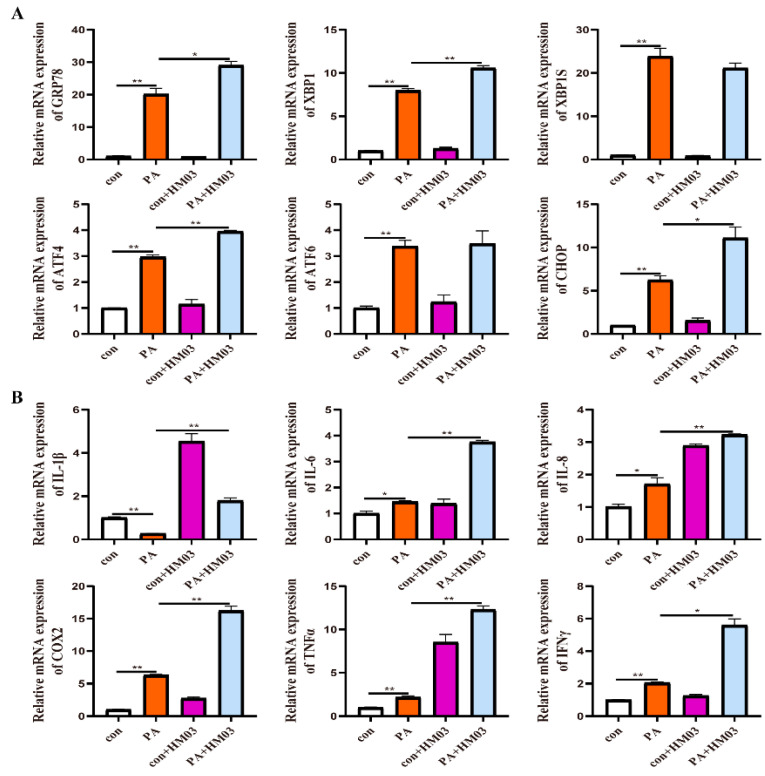
Effects of 10 μM HM03 treatment for 4 h after 500 μM PA incubation for 6 h on ER stress and inflammation in macrophages of large yellow croakers. (**A**) Relative mRNA expression of ER stress-related genes. (**B**) Relative mRNA expression of inflammatory genes. Values are presented as mean ± SEM (*n* = 3). * Significant difference (*p* < 0.05); ** highly significant difference (*p* < 0.01).

To confirm the effect of GRP78 on PA-induced ER stress and inflammation, 10 μM BiP and HM03 were incubated for 4 h before or after 500 μM PA incubation for 6 h. The results showed that the BiP treatment before PA incubation significantly downregulated the mRNA expression of *grp78*, *xbp1*, *xbp1s*, *chop*, *atf6*, and *atf4* compared with the PA treatment group (*p* < 0.05) (Figure 9A). Moreover, the proinflammatory genes, including *tnfα* and *ifnγ*, were significantly decreased (*p* < 0.05) (Figure 9B). The BiP treatment after PA incubation significantly downregulated the mRNA expression of *xbp1*, *xbp1s*, *chop*, *atf6*, and *atf4* compared with the PA treatment group (*p* < 0.05) (Figure 10A). Furthermore, the proinflammatory genes, including *il-8*, *cox2*, and *tnfα*, were significantly downregulated (*p* < 0.05) (Figure 10B). The HM03 treatment before PA incubation significantly upregulated the mRNA expression of *grp78*, *xbp1*, *chop*, and *atf4* compared with the PA treatment group (*p* < 0.05) (Figure 11A). Moreover, the proinflammatory genes, including *il-8*, *il-6*, *il-1β*, *cox2*, *tnfα*, and *ifnγ*, were significantly upregulated (*p* < 0.05) (Figure 11B). The HM03 treatment after PA incubation significantly upregulated the mRNA expression of *grp78*, *xbp1*, *chop*, and *atf4* compared with the PA treatment group (*p* < 0.05) (Figure 12A). Additionally, the proinflammatory genes, including *il-8*, *il-6*, *il-1β*, *cox2*, *tnfα*, and *ifnγ*, were significantly upregulated (*p* < 0.05) (Figure 12B). These results demonstrated that the BiP treatment could reduce the PA-induced ER stress and inflammatory response whereas the HM03 treatment could further induce the PA-induced ER stress and inflammatory response in the macrophages of large yellow croakers.

## 3. Discussion

The endoplasmic reticulum (ER) is the central organelle of the secretory pathways, which is the major place for synthesizing and exporting both lipids and proteins [32]. However, misfolded/unfolded proteins accumulate in the ER lumen, causing ER stress [33], which is a defense system for dealing with that situation. Previous studies have shown that ER stress is usually related to the inflammatory response [34,35]. Tunicamycin (TM) is a mixture of homologous nucleoside antibiotics, which can cause unfolded proteins to accumulate in the ER and induce ER stress. In the present study, TM incubation could significantly induce the expression of ER stress-related genes and proteins, which was consistent with previous studies [17,18,19,20]. Meanwhile, similar to previous studies [19,21,22,23], the TM treatment induced the expression of proinflammatory genes.

In this study, the data indicated that palmitic acid (PA) incubation increased ER stress, which was consistent with previous studies in mammals [36,37,38]. PA induces ER stress by increasing the content of saturated ER membrane phospholipids [39], perturbing ER calcium homeostasis [40] and modulating protein folding [41]. Furthermore, the PA treatment induced an inflammatory response, which was consistent with previous studies [24,27,28,29,30]. A study reported that dietary palm oil that contained a high level of PA could increase the inflammatory response by activating the TLR–NF-κB pathway [27]. Zhang [29] demonstrated that the IRE1α–XBP1s signaling pathway participated in PA-induced inflammation. Moreover, a previous study showed that PA induced inflammation via the TLR22–MAPK–PPARγ/Nrf2 pathway [30].

In order to figure out the relationship between glucose-regulated protein 78 (GRP78) and TM/PA-induced ER stress or inflammation, we used a GRP78 agonist (BiP inducer X) or inhibitor (HM03). In the present study, BiP inducer X (BiP) significantly upregulated the expression of GRP78 mRNA and the protein level. Similar to a previous study that observed that the rise of GRP78 mRNA by BiP was transient, the treatment for 4 h reached a peak, but the levels of GRP78 protein continued to upregulate up to 12 h [42]. Meanwhile, the BiP treatment increased the expression of ER stress-related genes and proteins, which was different from a previous study [42]. To be more specific, in that previous study, BiP preferentially increased GRP78 and slightly induced CHOP, calreticulin, and GRP94 mediated by the ATF6 pathway; however, BiP did not affect XBP1 or eif2α [42]. HM03 significantly decreased the GRP78 mRNA and protein levels; furthermore, the HM03 treatment increased the expression of ER stress-related genes.

In the present study, the BiP treatment after TM/PA incubation significantly decreased ER stress-related genes such as *xbp1s*, *atf6*, and *atf4* compared with the TM/PA treatment group. Consistent with previous studies that BiP is an effective ER stress inhibitor [43], BiP is known to significantly downregulate ER stress through an induced GRP78 mRNA level in animal models and in various cell lines [42,43,44,45]. Previous studies have indicated that the overexpression of GRP78 can reduce ER stress and increase thermogenesis [46]; moreover, the genetic overexpression of GRP78 was competent to reduce ER stress and to revert the metabolic and obese phenotype [47]. The results of the BiP treatment before PA incubation was consistent with the post-treatment of BiP. However, the BiP treatment before TM incubation significantly upregulated the mRNA expression of *xbp1*, *xbp1s*, *chop*, *atf6*, and *atf4* compared with the TM treatment group. These results may be because the BiP treatment could significantly increase ER stress-related genes, then TM incubation after the BiP treatment further induced the gene expression. Different from that under TM-induced ER stress, the overexpression of GRP78 through the BiP treatment could alleviate ER stress. These results also suggested that ER stress and inflammation induced by TM and PA were different. The HM03 treatment before or after TM/PA incubation significantly induced the expression of ER stress-related genes. Previous studies have shown that GRP78 siRNA increased the expression of UPR-induced genes such as *chop* [48]. An electron-microscopic analysis of the structure of intracellular organelles showed that the ER was greatly disorganized and expanded in the cells where GRP78 was downregulated [49]; in the cells where the GRP78 expression was downregulated by siRNA, the UPR pathways were activated.

Furthermore, the results from the present study indicated that the inflammatory response under different treatments was consistent with the ER stress response. GRP78 has been indicated to be anti-inflammatory and promote the resolution of inflammation [12,13,14]. A previous study revealed that GRP78 mediated the endocytosis of TLR4 by targeting CD14, which is conducive to the regression of an inflammatory response [13]. Moreover, an adoptive GRP78–DC transfer was essential to resolve the inflammatory response in NOD mice [14]. The results suggested that the inflammatory response may be regulated by GRP78 on the one hand and be regulated by ER stress on the other hand. 

In conclusion, we found that GRP78 could regulate TM/PA-induced ER stress or inflammation. To be more specific, the overexpression of GRP78 could reduce the TM/PA-induced ER stress and inflammatory response. These results provide a novel idea to explain the relationship between GRP78 and TM/PA-induced ER stress or inflammation in large yellow croakers.

## 4. Materials and Methods

### 4.1. Cell Culture and Treatment

The macrophage line of large yellow croakers was obtained from our colleagues [50]. The macrophages were seeded into 6-well plates at a density of 2.0 × 10^6^ cells/well and were maintained in a DMEM/F12 medium (BI, Kibbutz Beit-Haemek, Israel) containing 15% FBS (BI, Kibbutz Beit-Haemek, Israel) and antibiotics (Solarbio, Beijing, China) in a 5% CO_2_ atmosphere at 28 °C. To figure out the effects of TM on ER stress and the inflammatory response in the macrophages, the cells were incubated with 1 μM TM for different time points. The control group was treated with DMSO of the same concentration (Solarbio, Beijing, China). The cells were incubated with PA to confirm the effects of PA on ER stress and the inflammatory response in the macrophages. PA (Sigma, St. Louis, MO, USA) was supplemented to the cells with the forms of BSA/fatty acid complexes at 500 μM for different time points. The solvent group was set as the control group. To investigate the role of GRP78 in TM/PA-induced ER stress and the inflammatory response, a GRP78 agonist (BiP inducer X; MCE, Jersey City, NJ, USA) and a GRP78 inhibitor (HM03; MCE, Jersey City, NJ, USA) were treated with a concentration of 10 μM for 4 h before or after the TM/PA treatment. After incubation, the cells were lysed in the wells and harvested for the further analysis.

### 4.2. RNA Extraction and RT-qPCR

The harvested macrophage cells were added to a Trizol reagent (Takara, Dalian, China). The total RNA was then extracted according to the manufacturer’s instructions. The quality of RNA was detected by electrophoresis using 1.2% denatured agarose gel. The RNA was reversed to cDNA by a PrimeScript RT reagent kit (Takara, Dalian, China) following the manufacturer’s protocol. The mRNA expression levels were quantified in a quantitative thermal cycler (Bio-rad, Hercules, CA, USA) with an SYBR Green real-time PCR kit (Takara, Dalian, China). The normalization of the gene expression in different tissues of large yellow croakers was detected by *rpl17*, *gapdh*, and *β-actin* to test their suitability. Among all treatments, no significant differences in *β-actin* expression were detected, suggesting that *β-actin* could be used as a reference gene. Further, the mRNA expression of each gene was normalized to that of *β-actin* using the 2^−ΔΔct^ method [51].

### 4.3. Western Blot

Western blotting was carried out following the methods in previous studies [30,52,53]. The primary antibodies against JNK1/2 (Cat. No. 9252), p-JNK1/2 (Cat. No. 4668), ERK1/2 (Cat. No. 4695), p-ERK1/2 (Cat. No. 4370), p-p38 (Cat. No. 9215), GRP78 (Cat. No. 3177), XBP1s (Cat. No. 12782), PERK (Cat. No. 5683), p-eif2α (Cat. No. 9721), eif2α (Cat. No. 9722), and CHOP (Cat. No. 5554) were purchased from Cell Signaling Technology (Boston, MA, USA). The antibody against p-PERK (Cat. No. abs137056) was purchased from Absin Biotechnology (China). GAPDH (AF1186) and HRP-conjugated secondary antibodies (A0208) were obtained from Beyotime. The target proteins were quantified using ImageJ software (National Institutes of Health, Bethesda, MD, USA).

### 4.4. Statistical Analysis

All data were presented as means ± SEM. All data were subjected to independent *t*-tests with the help of SPSS 19.0. A *p* < 0.05 was applied as a significant difference and a *p* < 0.01 as a highly significant difference.

## Data Availability

Data is contained within the article.
